# REM sleep behaviour disorder (RBD): risk for Parkinsonism and executive dysfunction in elderly

**DOI:** 10.18632/oncotarget.26417

**Published:** 2018-12-04

**Authors:** Stefanie Lerche, Kathrin Brockmann

**Affiliations:** Stefanie Lerche: Center of Neurology, Department of Neurodegeneration and Hertie-Institute for Clinical Brain Research, University of Tuebingen, Tuebingen, Germany; German Center for Neurodegenerative Diseases, University of Tuebingen, Tuebingen, Germany

**Keywords:** RBD, executive dysfunction, Parkinsonism, aging, Gerotarget

Parkinson’s disease (PD) is a neurodegenerative disorder which was originally conceptualized as a motor disease, and its diagnosis is still based on the core motor features bradykinesia, resting tremor, and rigidity. Remarkably, 40-60% of the dopaminergic neurons are already degenerated at the time when motor symptoms allow clinical diagnosis. This timespan in which neurodegeneration is proceeding without leading to classical motor symptoms that allow a diagnosis of clinical PD is termed “prodromal phase of PD”.

During this prodromal phase, a variety of clinical non-motor symptoms (NMS) may occur. These NMS are heterogeneous and typically include, among others, constipation, hyposmia, sleep disorders (rapid eye movement behavior disorder (RBD) and excessive daytime sleepiness) and neuropsychiatric symptoms such as depression. In contrast to other NMS, cognitive symptoms are less well understood as a prodromal sign of PD. It seems plausible that cognitive changes may also occur in the prodromal phase of the disease, given that a substantial proportion of newly diagnosed PD patients exhibits cognitive symptoms, especially executive dysfunction. This is typically assigned to dopaminergic dysfunction in the “cognitive loop” of the frontostriatal circuits that projects from the basal ganglia to the dorsolateral prefrontal cortex [1].

Disturbed sleep is a common feature in neurodegenerative diseases and can greatly affect quality of life, productivity and health care of patients and spouses. In this context, sleep architecture of patients with cognitive impairment, including patients with Alzheimer disease, PD and Dementia with Lewy bodies (DLB) differs compared to healthy control individuals [2]. It is well known that patients with alpha-synucleinopathies such as PD and DLB often show RBD during the disease course (PD 16-47%, DLB 80%) [3]. Further, RBD has been shown to be one of the strongest prodromal markers for the development of synucleinopathies as over 80% of people with RBD eventually develop a synucleinopathy within 10 years [4] and a decline in cognitive function is frequent in RBD patients.

However, only a few studies investigating patients with RBD and a subsequent established PD diagnosis also providing data on cognitive functions are available. One study tested prodromal PD markers in a 10-year prospective cohort and compared 41 patients with idiopathic RBD who were subsequently diagnosed with PD to 48 RBD patients who had not developed PD at follow-up after a mean time of 5.4 ± 2.9 years [5]. No clear evidence was found that the presence of cognitive impairment at baseline could predict the conversion to PD. However, although the difference was not significant, 70% of RBD patients with subsequent PD diagnosis presented with cognitive impairment at baseline, compared to 50% of RBD patients without subsequent PD diagnosis. Unfortunately, comparisons of cognitive data for the two RBD groups were not reported in detail for single domains. In another study, 24 patients with RBD were included and three of them developed PD at follow-up after a mean time of 26.3 months [6]. Although an elaborate neuropsychological test battery was used assessing global cognitive state, executive function, memory, and visuospatial function, cognitive functions at baseline did not predict the development of PD. The significance of the findings of this study is generally limited by the small sample size.

Based on these findings and since PD patients primarily suffer from executive dysfunction, we hypothesized that individuals with RBD may show impairment in the executive domain. We investigated 1145 healthy elderly cross-sectionally and of those a subgroup of 544 longitudinally over 6 years [7]. Participants were investigated neurologically as well as with different questionnaires for NMS and with a comprehensive neuropsychological test battery. Participants with RBD present with reduced executive performance compared to non-RBD participants cross-sectionally with enhanced deterioration longitudinally. After a mean follow-up of 4.6 years 4.4% of the RBD group developed PD whereas only 0.8% of the non-RBD group converted to PD over time.

So far, no preventive and neuroprotective therapies for PD exist. The understanding of NMS in the prodromal phase of the disease may help to reliably identify at-risk individuals who are the optimal candidates for neuroprotective trials. Given the need to develop neuroprotective therapies in PD preventing cognitive decline (and other symptoms), the understanding of prodromal cognitive changes seems to be of great importance.
